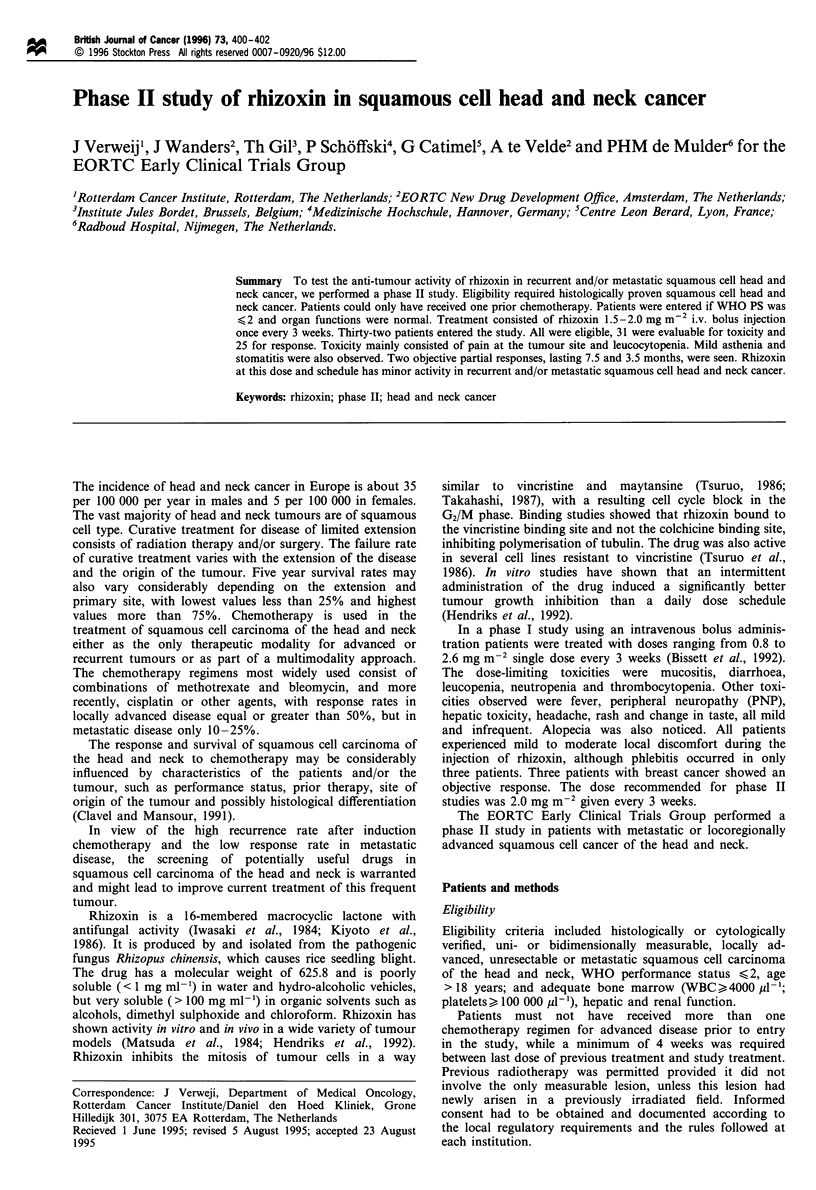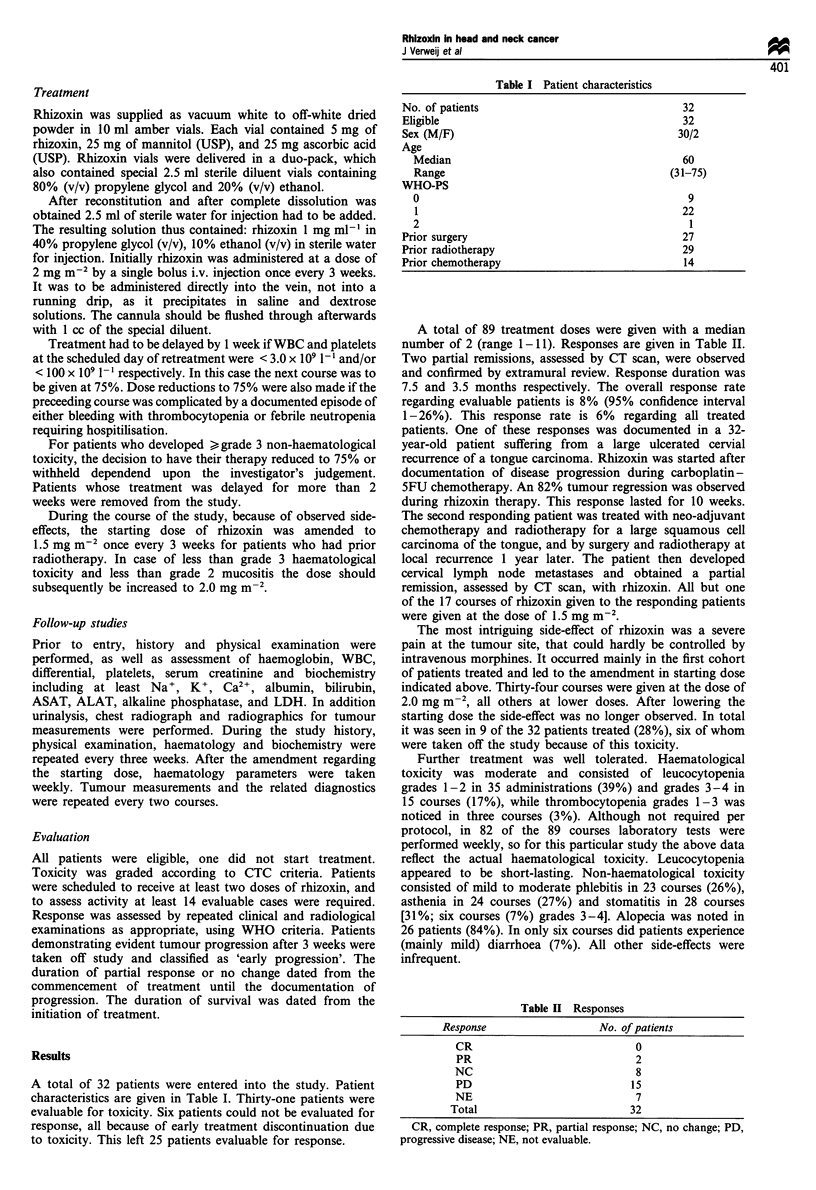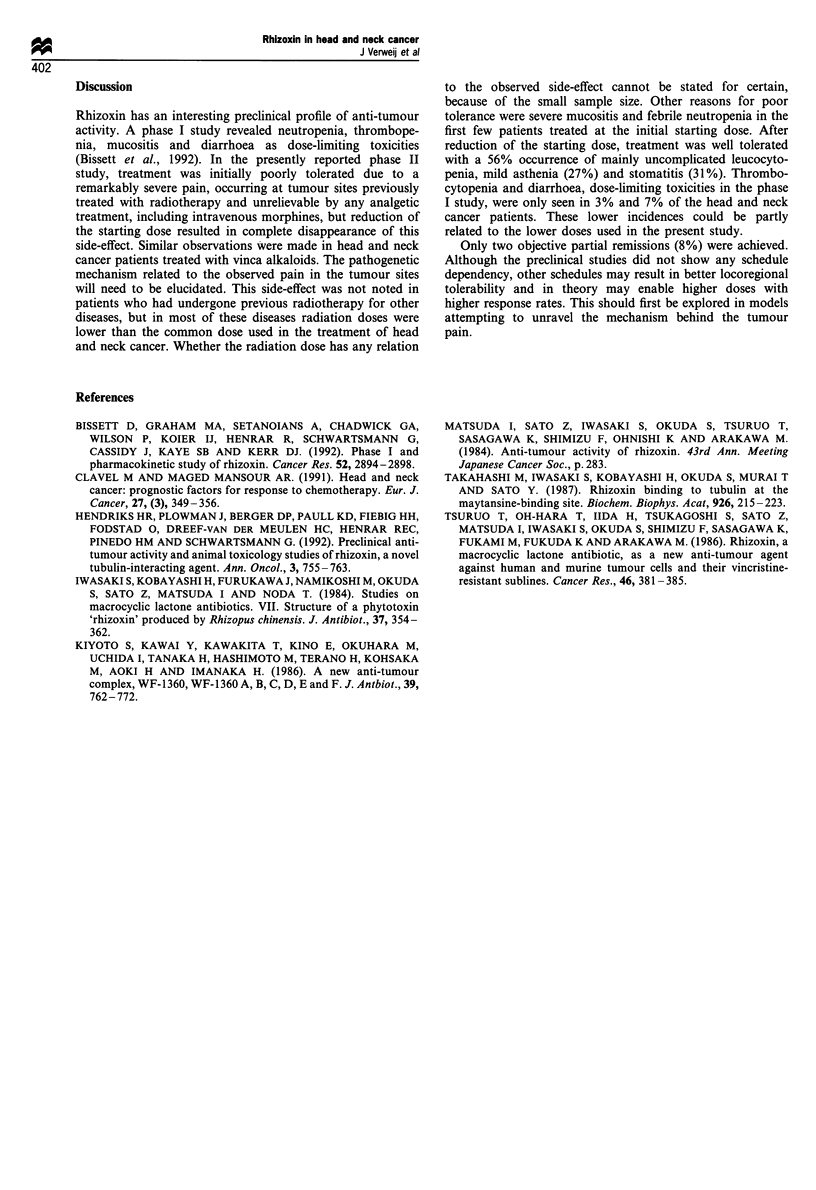# Phase II study of rhizoxin in squamous cell head and neck cancer. The EORTC Early Clinical Trials Group.

**DOI:** 10.1038/bjc.1996.69

**Published:** 1996-02

**Authors:** J. Verweij, J. Wanders, T. Gil, P. Schöffski, G. Catimel, A. te Velde, P. H. de Mulder

**Affiliations:** Rotterdam Cancer Institute, The Netherlands.

## Abstract

To test the anti-tumour activity of rhizoxin in recurrent and/or metastatic squamous cell head and neck cancer, we performed a phase II study. Eligibility required histologically proven squamous cell head and neck cancer. Patients could only have received one prior chemotherapy. Patients were entered if WHO PS was < or = 2 and organ functions were normal. Treatment consisted of rhizoxin 1.5-2.0 mg m-2 i.v. bolus injection once every 3 weeks. Thirty-two patients entered the study. All were eligible, 31 were evaluable for toxicity and 25 for response. Toxicity mainly consisted of pain at the tumour site and leucocytopenia. Mild asthenia and stomatitis were also observed. Two objective partial responses, lasting 7.5 and 3.5 months, were seen. Rhizoxin at this dose and schedule has minor activity in recurrent and/or metastatic squamous cell head and neck cancer.


British Journal of Cancer (1996) 73, 400-402
© 1996 Stockton Press All rights reserved 0007-0920/96 $12.00

### Phase II study of rhizoxin in squamous cell head and neck cancer

J Verweij[1], J Wanders[2], Th Gil[3], P Schöffski[4], G Catimel[5], A te Velde[2] and PHM de Mulder[6] for the EORTC Early Clinical Trials Group

*[1]Rotterdam Cancer Institute, Rotterdam, The Netherlands; [2]EORTC New Drug Development Office, Amsterdam, The Netherlands; [3]Institute Jules Bordet, Brussels, Belgium; [4]Medizinische Hochschule, Hannover, Germany; [5]Centre Leon Berard, Lyon, France; [6]Radboud Hospital, Nijmegen, The Netherlands.*

**Summary** To test the anti-tumour activity of rhizoxin in recurrent and/or metastatic squamous cell head and neck cancer, we performed a phase II study. Eligibility required histologically proven squamous cell head and neck cancer. Patients could only have received one prior chemotherapy. Patients were entered if WHO PS was $\leqslant 2$ and organ functions were normal. Treatment consisted of rhizoxin 1.5–2.0 mg m$^{-2}$ i.v. bolus injection once every 3 weeks. Thirty-two patients entered the study. All were eligible, 31 were evaluable for toxicity and 25 for response. Toxicity mainly consisted of pain at the tumour site and leucocytopenia. Mild asthenia and stomatitis were also observed. Two objective partial responses, lasting 7.5 and 3.5 months, were seen. Rhizoxin at this dose and schedule has minor activity in recurrent and/or metastatic squamous cell head and neck cancer.

**Keywords:** rhizoxin; phase II; head and neck cancer

The incidence of head and neck cancer in Europe is about 35 per 100 000 per year in males and 5 per 100 000 in females. The vast majority of head and neck tumours are of squamous cell type. Curative treatment for disease of limited extension consists of radiation therapy and/or surgery. The failure rate of curative treatment varies with the extension of the disease and the origin of the tumour. Five year survival rates may also vary considerably depending on the extension and primary site, with lowest values less than 25% and highest values more than 75%. Chemotherapy is used in the treatment of squamous cell carcinoma of the head and neck either as the only therapeutic modality for advanced or recurrent tumours or as part of a multimodality approach. The chemotherapy regimens most widely used consist of combinations of methotrexate and bleomycin, and more recently, cisplatin or other agents, with response rates in locally advanced disease equal or greater than 50%, but in metastatic disease only 10–25%.

The response and survival of squamous cell carcinoma of the head and neck to chemotherapy may be considerably influenced by characteristics of the patients and/or the tumour, such as performance status, prior therapy, site of origin of the tumour and possibly histological differentiation (Clavel and Mansour, 1991).

In view of the high recurrence rate after induction chemotherapy and the low response rate in metastatic disease, the screening of potentially useful drugs in squamous cell carcinoma of the head and neck is warranted and might lead to improve current treatment of this frequent tumour.

Rhizoxin is a 16-membered macrocyclic lactone with antifungal activity (Iwasaki *et al.*, 1984; Kiyoto *et al.*, 1986). It is produced by and isolated from the pathogenic fungus *Rhizopus chinensis*, which causes rice seedling blight. The drug has a molecular weight of 625.8 and is poorly soluble (< 1 mg ml$^{-1}$) in water and hydro-alcoholic vehicles, but very soluble (> 100 mg ml$^{-1}$) in organic solvents such as alcohols, dimethyl sulphoxide and chloroform. Rhizoxin has shown activity *in vitro* and *in vivo* in a wide variety of tumour models (Matsuda *et al.*, 1984; Hendriks *et al.*, 1992). Rhizoxin inhibits the mitosis of tumour cells in a way similar to vincristine and maytansine (Tsuruo, 1986; Takahashi, 1987), with a resulting cell cycle block in the $G_2/M$ phase. Binding studies showed that rhizoxin bound to the vincristine binding site and not the colchicine binding site, inhibiting polymerisation of tubulin. The drug was also active in several cell lines resistant to vincristine (Tsuruo *et al.*, 1986). *In vitro* studies have shown that an intermittent administration of the drug induced a significantly better tumour growth inhibition than a daily dose schedule (Hendriks *et al.*, 1992).

In a phase I study using an intravenous bolus administration patients were treated with doses ranging from 0.8 to 2.6 mg m$^{-2}$ single dose every 3 weeks (Bissett *et al.*, 1992). The dose-limiting toxicities were mucositis, diarrhoea, leucopenia, neutropenia and thrombocytopenia. Other toxicities observed were fever, peripheral neuropathy (PNP), hepatic toxicity, headache, rash and change in taste, all mild and infrequent. Alopecia was also noticed. All patients experienced mild to moderate local discomfort during the injection of rhizoxin, although phlebitis occurred in only three patients. Three patients with breast cancer showed an objective response. The dose recommended for phase II studies was 2.0 mg m$^{-2}$ given every 3 weeks.

The EORTC Early Clinical Trials Group performed a phase II study in patients with metastatic or locoregionally advanced squamous cell cancer of the head and neck.

### Patients and methods

### *Eligibility*

Eligibility criteria included histologically or cytologically verified, uni- or bidimensionally measurable, locally advanced, unresectable or metastatic squamous cell carcinoma of the head and neck, WHO performance status $\leqslant 2$, age > 18 years; and adequate bone marrow (WBC$\geqslant$4000 $\mu$l$^{-1}$; platelets$\geqslant$100 000 $\mu$l$^{-1}$), hepatic and renal function.

Patients must not have received more than one chemotherapy regimen for advanced disease prior to entry in the study, while a minimum of 4 weeks was required between last dose of previous treatment and study treatment. Previous radiotherapy was permitted provided it did not involve the only measurable lesion, unless this lesion had newly arisen in a previously irradiated field. Informed consent had to be obtained and documented according to the local regulatory requirements and the rules followed at each institution.

Correspondence: J Verweji, Department of Medical Oncology, Rotterdam Cancer Institute/Daniel den Hoed Kliniek, Grone Hilledijk 301, 3075 EA Rotterdam, The Netherlands
Recieved 1 June 1995; revised 5 August 1995; accepted 23 August 1995

*Treatment*

Rhizoxin was supplied as vacuum white to off-white dried powder in 10 ml amber vials. Each vial contained 5 mg of rhizoxin, 25 mg of mannitol (USP), and 25 mg ascorbic acid (USP). Rhizoxin vials were delivered in a duo-pack, which also contained special 2.5 ml sterile diluent vials containing 80% (v/v) propylene glycol and 20% (v/v) ethanol.

After reconstitution and after complete dissolution was obtained 2.5 ml of sterile water for injection had to be added. The resulting solution thus contained: rhizoxin 1 mg $ml^{-1}$ in 40% propylene glycol (v/v), 10% ethanol (v/v) in sterile water for injection. Initially rhizoxin was administered at a dose of 2 mg $m^{-2}$ by a single bolus i.v. injection once every 3 weeks. It was to be administered directly into the vein, not into a running drip, as it precipitates in saline and dextrose solutions. The cannula should be flushed through afterwards with 1 cc of the special diluent.

Treatment had to be delayed by 1 week if WBC and platelets at the scheduled day of retreatment were $< 3.0 \times 10^9\ l^{-1}$ and/or $< 100 \times 10^9\ l^{-1}$ respectively. In this case the next course was to be given at 75%. Dose reductions to 75% were also made if the preceeding course was complicated by a documented episode of either bleeding with thrombocytopenia or febrile neutropenia requiring hospitilisation.

For patients who developed $\geqslant$grade 3 non-haematological toxicity, the decision to have their therapy reduced to 75% or withheld dependend upon the investigator's judgement. Patients whose treatment was delayed for more than 2 weeks were removed from the study.

During the course of the study, because of observed side-effects, the starting dose of rhizoxin was amended to 1.5 mg $m^{-2}$ once every 3 weeks for patients who had prior radiotherapy. In case of less than grade 3 haematological toxicity and less than grade 2 mucositis the dose should subsequently be increased to 2.0 mg $m^{-2}$.

*Follow-up studies*

Prior to entry, history and physical examination were performed, as well as assessment of haemoglobin, WBC, differential, platelets, serum creatinine and biochemistry including at least $Na^+$, $K^+$, $Ca^{2+}$, albumin, bilirubin, ASAT, ALAT, alkaline phosphatase, and LDH. In addition urinalysis, chest radiograph and radiographics for tumour measurements were performed. During the study history, physical examination, haematology and biochemistry were repeated every three weeks. After the amendment regarding the starting dose, haematology parameters were taken weekly. Tumour measurements and the related diagnostics were repeated every two courses.

*Evaluation*

All patients were eligible, one did not start treatment. Toxicity was graded according to CTC criteria. Patients were scheduled to receive at least two doses of rhizoxin, and to assess activity at least 14 evaluable cases were required. Response was assessed by repeated clinical and radiological examinations as appropriate, using WHO criteria. Patients demonstrating evident tumour progression after 3 weeks were taken off study and classified as 'early progression'. The duration of partial response or no change dated from the commencement of treatment until the documentation of progression. The duration of survival was dated from the initiation of treatment.

### Results

A total of 32 patients were entered into the study. Patient characteristics are given in Table I. Thirty-one patients were evaluable for toxicity. Six patients could not be evaluated for response, all because of early treatment discontinuation due to toxicity. This left 25 patients evaluable for response.

**Table I** Patient characteristics

| | |
|---|---|
| No. of patients | 32 |
| Eligible | 32 |
| Sex (M/F) | 30/2 |
| Age | |
| Median | 60 |
| Range | (31-75) |
| WHO-PS | |
| 0 | 9 |
| 1 | 22 |
| 2 | 1 |
| Prior surgery | 27 |
| Prior radiotherapy | 29 |
| Prior chemotherapy | 14 |

A total of 89 treatment doses were given with a median number of 2 (range 1–11). Responses are given in Table II. Two partial remissions, assessed by CT scan, were observed and confirmed by extramural review. Response duration was 7.5 and 3.5 months respectively. The overall response rate regarding evaluable patients is 8% (95% confidence interval 1–26%). This response rate is 6% regarding all treated patients. One of these responses was documented in a 32-year-old patient suffering from a large ulcerated cervial recurrence of a tongue carcinoma. Rhizoxin was started after documentation of disease progression during carboplatin–5FU chemotherapy. An 82% tumour regression was observed during rhizoxin therapy. This response lasted for 10 weeks. The second responding patient was treated with neo-adjuvant chemotherapy and radiotherapy for a large squamous cell carcinoma of the tongue, and by surgery and radiotherapy at local recurrence 1 year later. The patient then developed cervical lymph node metastases and obtained a partial remission, assessed by CT scan, with rhizoxin. All but one of the 17 courses of rhizoxin given to the responding patients were given at the dose of 1.5 mg $m^{-2}$.

The most intriguing side-effect of rhizoxin was a severe pain at the tumour site, that could hardly be controlled by intravenous morphines. It occurred mainly in the first cohort of patients treated and led to the amendment in starting dose indicated above. Thirty-four courses were given at the dose of 2.0 mg $m^{-2}$, all others at lower doses. After lowering the starting dose the side-effect was no longer observed. In total it was seen in 9 of the 32 patients treated (28%), six of whom were taken off the study because of this toxicity.

Further treatment was well tolerated. Haematological toxicity was moderate and consisted of leucocytopenia grades 1–2 in 35 administrations (39%) and grades 3–4 in 15 courses (17%), while thrombocytopenia grades 1–3 was noticed in three courses (3%). Although not required per protocol, in 82 of the 89 courses laboratory tests were performed weekly, so for this particular study the above data reflect the actual haematological toxicity. Leucocytopenia appeared to be short-lasting. Non-haematological toxicity consisted of mild to moderate phlebitis in 23 courses (26%), asthenia in 24 courses (27%) and stomatitis in 28 courses [31%; six courses (7%) grades 3–4]. Alopecia was noted in 26 patients (84%). In only six courses did patients experience (mainly mild) diarrhoea (7%). All other side-effects were infrequent.

**Table II** Responses

| *Response* | *No. of patients* |
|---|---|
| CR | 0 |
| PR | 2 |
| NC | 8 |
| PD | 15 |
| NE | 7 |
| Total | 32 |

CR, complete response; PR, partial response; NC, no change; PD, progressive disease; NE, not evaluable.

### Discussion

Rhizoxin has an interesting preclinical profile of anti-tumour activity. A phase I study revealed neutropenia, thrombopenia, mucositis and diarrhoea as dose-limiting toxicities (Bissett *et al.*, 1992). In the presently reported phase II study, treatment was initially poorly tolerated due to a remarkably severe pain, occurring at tumour sites previously treated with radiotherapy and unrelievable by any analgetic treatment, including intravenous morphines, but reduction of the starting dose resulted in complete disappearance of this side-effect. Similar observations were made in head and neck cancer patients treated with vinca alkaloids. The pathogenetic mechanism related to the observed pain in the tumour sites will need to be elucidated. This side-effect was not noted in patients who had undergone previous radiotherapy for other diseases, but in most of these diseases radiation doses were lower than the common dose used in the treatment of head and neck cancer. Whether the radiation dose has any relation to the observed side-effect cannot be stated for certain, because of the small sample size. Other reasons for poor tolerance were severe mucositis and febrile neutropenia in the first few patients treated at the initial starting dose. After reduction of the starting dose, treatment was well tolerated with a 56% occurrence of mainly uncomplicated leucocytopenia, mild asthenia (27%) and stomatitis (31%). Thrombocytopenia and diarrhoea, dose-limiting toxicities in the phase I study, were only seen in 3% and 7% of the head and neck cancer patients. These lower incidences could be partly related to the lower doses used in the present study.

Only two objective partial remissions (8%) were achieved. Although the preclinical studies did not show any schedule dependency, other schedules may result in better locoregional tolerability and in theory may enable higher doses with higher response rates. This should first be explored in models attempting to unravel the mechanism behind the tumour pain.

### References

BISSETT D, GRAHAM MA, SETANOIANS A, CHADWICK GA, WILSON P, KOIER IJ, HENRAR R, SCHWARTSMANN G, CASSIDY J, KAYE SB AND KERR DJ. (1992). Phase I and pharmacokinetic study of rhizoxin. *Cancer Res.* **52,** 2894–2898.

CLAVEL M AND MAGED MANSOUR AR. (1991). Head and neck cancer: prognostic factors for response to chemotherapy. *Eur. J. Cancer*, **27, (3),** 349–356.

HENDRIKS HR, PLOWMAN J, BERGER DP, PAULL KD, FIEBIG HH, FODSTAD O, DREEF-VAN DER MEULEN HC, HENRAR REC, PINEDO HM AND SCHWARTSMANN G. (1992). Preclinical antitumour activity and animal toxicology studies of rhizoxin, a novel tubulin-interacting agent. *Ann. Oncol.*, **3,** 755–763.

IWASAKI S, KOBAYASHI H, FURUKAWA J, NAMIKOSHI M, OKUDA S, SATO Z, MATSUDA I AND NODA T. (1984). Studies on macrocyclic lactone antibiotics. VII. Structure of a phytotoxin 'rhizoxin' produced by *Rhizopus chinensis*. *J. Antibiot.*, **37,** 354–362.

KIYOTO S, KAWAI Y, KAWAKITA T, KINO E, OKUHARA M, UCHIDA I, TANAKA H, HASHIMOTO M, TERANO H, KOHSAKA M, AOKI H AND IMANAKA H. (1986). A new anti-tumour complex, WF-1360, WF-1360 A, B, C, D, E and F. *J. Antbiot.*, **39,** 762–772.

MATSUDA I, SATO Z, IWASAKI S, OKUDA S, TSURUO T, SASAGAWA K, SHIMIZU F, OHNISHI K AND ARAKAWA M. (1984). Anti-tumour activity of rhizoxin. *43rd Ann. Meeting Japanese Cancer Soc.*, p. 283.

TAKAHASHI M, IWASAKI S, KOBAYASHI H, OKUDA S, MURAI T AND SATO Y. (1987). Rhizoxin binding to tubulin at the maytansine-binding site. *Biochem. Biophys. Acat*, **926,** 215–223.

TSURUO T, OH-HARA T, IIDA H, TSUKAGOSHI S, SATO Z, MATSUDA I, IWASAKI S, OKUDA S, SHIMIZU F, SASAGAWA K, FUKAMI M, FUKUDA K AND ARAKAWA M. (1986). Rhizoxin, a macrocyclic lactone antibiotic, as a new anti-tumour agent against human and murine tumour cells and their vincristine-resistant sublines. *Cancer Res.*, **46,** 381–385.